# MitoLoc: A method for the simultaneous quantification of mitochondrial network morphology and membrane potential in single cells

**DOI:** 10.1016/j.mito.2015.07.001

**Published:** 2015-09

**Authors:** Jakob Vowinckel, Johannes Hartl, Richard Butler, Markus Ralser

**Affiliations:** a Dept. of Biochemistry and Cambridge Systems Biology Centre, University of Cambridge, 80 Tennis Court Road, Cambridge, UK; b The Wellcome Trust/Cancer Research UK Gurdon Institute, University of Cambridge, Tennis Court Road, Cambridge, UK; c The Francis Crick Institute, Mill Hill Laboratory, London NW7 1AA, UK

**Keywords:** Mitochondrial morphology, Membrane potential, Super resolution microscopy, Quantitative microscopy

## Abstract

Mitochondria assemble into flexible networks. Here we present a simple method for the simultaneous quantification of mitochondrial membrane potential and network morphology that is based on computational co-localisation analysis of differentially imported fluorescent marker proteins. Established in, but not restricted to, *Saccharomyces cerevisiae*, MitoLoc reproducibly measures changes in membrane potential induced by the uncoupling agent CCCP, by oxidative stress, in respiratory deficient cells, and in ∆*fzo1*, ∆*ref2*, and ∆*dnm1* mutants that possess fission and fusion defects. In combination with super-resolution images, MitoLoc uses 3D reconstruction to calculate six geometrical classifiers which differentiate network morphologies in ∆*fzo1*, ∆*ref2*, and ∆*dnm1* mutants, under oxidative stress and in cells lacking mtDNA, even when the network is fragmented to a similar extent. We find that mitochondrial fission and a decline in membrane potential do regularly, but not necessarily, co-occur. MitoLoc hence simplifies the measurement of mitochondrial membrane potential in parallel to detect morphological changes in mitochondrial networks. Marker plasmid open-source software as well as the mathematical procedures are made openly available.

## Highlights

• MitoLoc is a workflow for measuring changes mitochondrial membrane potential and morphology in parallel.
• Pixel-by pixel co-localisation of two fluorescent reporters is used to detect changes in mitochondrial membrane potential.
• Automated 3D reconstruction of super resolution images to calculate mitochondrial morphological classifiers
• We make fluorescent markers, mathematical procedures and software openly available.
• We demonstrate the application of MitoLoc on several examples.

## 1 Introduction

Mitochondria are central metabolic organelles for eukaryotic cells. They are required in energetic and biosynthetic metabolism and as such are crucial for cellular growth and survival. Potentially descending from Archean symbiotic proteobacteria, most eukaryotic mitochondria carry their own genetic material, host the tricarboxylic acid (TCA) cycle, are required for the assembly of iron–sulphur clusters, the respiratory chain, and are integral part of the apoptotic machinery (Frazzon et al., 2002; Madeo et al., 2009; Schatz, 1995; Veatch et al., 2009; Wallace, 1999). This central role requires mitochondria to constantly adapt to changes in cellular physiology. The presence of a mitochondrial membrane potential (MMP) constituted by the electron transport chain is the main determinant of organelle functionality (Dimroth et al., 2000; Mitchell, 1966). The MMP is required for mitochondrial ATP production by the F_1_F_0_ ATPase (Complex V), for import of mitochondrial proteins, and is essential for the maintenance of mitochondrial ion homeostasis (O'Rourke et al., 2005).

Size, shape, position and volume of mitochondrial networks are sensitive to environmental and physiological requirements. Under metabolically preferential conditions, single mitochondrial units fuse into large structures that can span the cell as single, tubular entities (Friedman and Nunnari, 2014). When shifted from fermentative to oxidative metabolism for example, mitochondrial volume increases drastically (Egner et al., 2002). Other perturbations can lead to single, condensed mitochondrial structures. In stress situations, such as changes in the availability of nutrients or exposure to mitochondrial damaging agents (Rafelski, 2013), loss of the MMP leads to fission of the mitochondrial network into smaller units, which facilitates the protection of mitochondria, or when damage is excessive, their degradation by the process of mitophagy (Breitenbach et al., 2014; Kubli and Gustafsson, 2012).

As MMP and mitochondrial fragmentation status are not necessarily coupled to each other (Bajić et al., 2013; Konno and Kako, 1991), both parameters need to measured in order to evaluate the physiological status of mitochondrial functionality. Measurement of MMP in intact cells is traditionally achieved using the MMP-dependent import of dyes that accumulate in intact mitochondria and are subsequently detected using flow cytometry or microscopy. Dyes in regular use include the single wavelength probes Rhodamine-123 and DiOC_6_, or recently, metachromatic dyes like JC-1 in combination with flow cytometry (Cossarizza et al., 1993; Scaduto and Grotyohann, 1999; Troiano et al., 2007).

Although the importance of mitochondrial morphology and function are equally well accepted, these are much more difficult to assess in a quantitative manner, so that quantitative analysis allowing comparison of network morphologies between different laboratories and studies has so far been hampered by a shortage of easily applicable techniques. Indeed, most current studies rely on qualitative categorisation of mitochondrial morphologies based on visual inspection of mitochondrial structures and fragmentation in fluorescence microscopy images. The characterisation of mitochondrial volume and morphology is thus easily biassed and dependent on the experience of the experimenter. Recently however, elaborate computational analysis of mitochondrial structures has shown that mitochondrial networks can in principle be described in numerical terms (Lin et al., 2010; Peng et al., 2011; Sukhorukov et al., 2012). If easily available, such metrics would thus reduce the subjectivity in assessing mitochondrial morphologies.

For this reason, we established a broadly applicable and simple workflow for the quantitative assessment of mitochondrial network morphology. We combine this with a new method to determine the membrane potential in parallel. The system is based on wide-field fluorescence microscopy imaging of two mitochondrial localised fluorescent proteins, one of which is MMP-dependently and the other one MMP-independently imported. Pixel by pixel co-localisation analysis allows a highly reproducible assessment of the MMP. When used in combination with super-resolution microscopy, the system reconstructs mitochondrial networks in 3D, and we propose a computational analysis strategy able to extract a range of geometrical features of the mitochondrial network in quantitative, objective terms.

## 2 Results

### 2.1 Construction of a dual-reporter system for measurement of mitochondrial membrane potential using co-localisation analysis

**Fig. 1:**
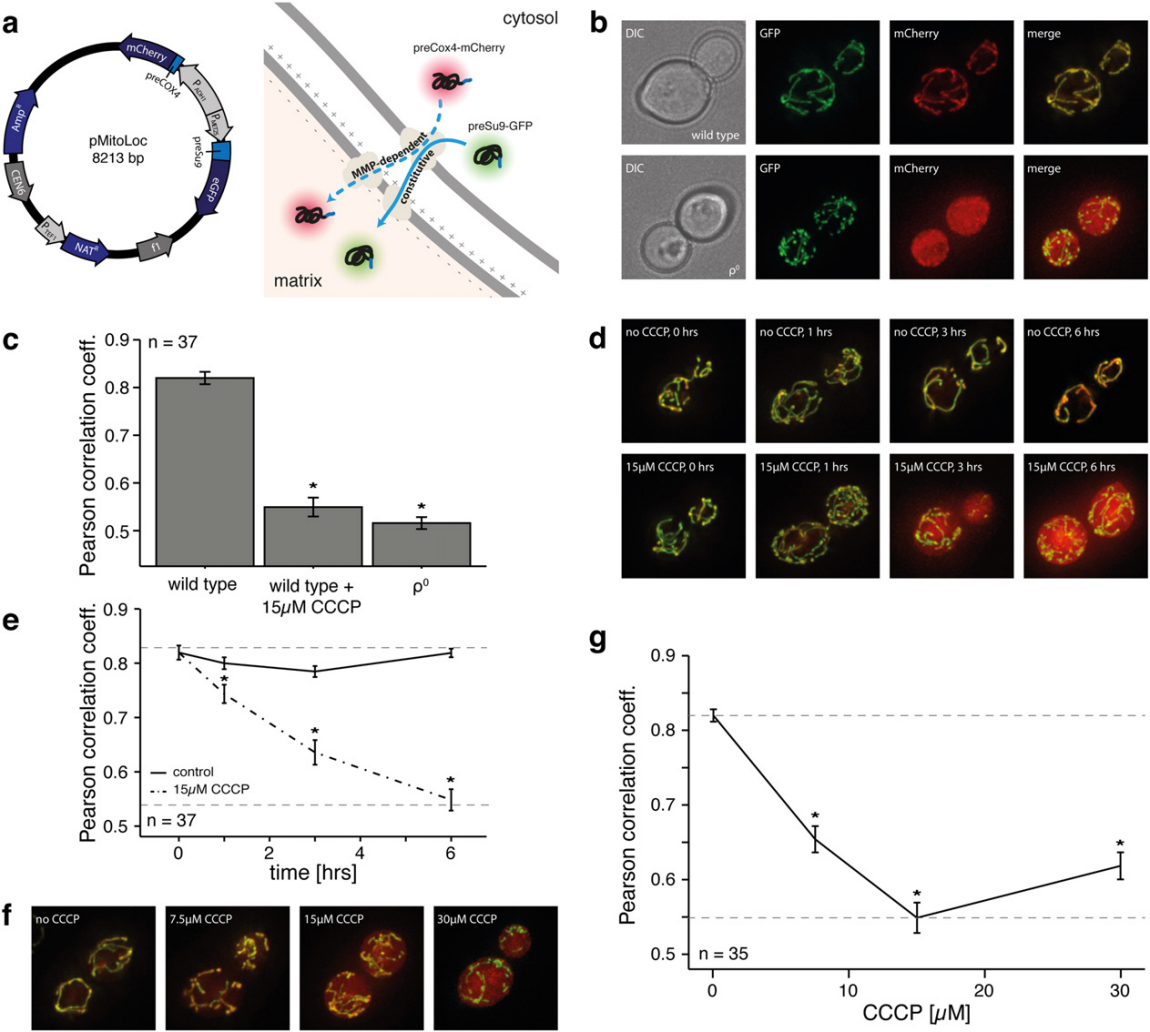
Quantification of the mitochondrial membrane potential by co-correlation analysis of differentially imported mitochondrial proteins. (a) (left) Plasmid map of pMitoLoc and (right) schematic outline of the MMP-dependent and -independent mitochondrial import of preCOX4-mCherry and preSU9-GFP. (b) Wide-field microscopy images of YSBN1 wild type and respiratory-deficient ρ^0^ yeast cells carrying pMitoLoc. Mitochondrial structures are detected by preSU9-GFP (green). In typical wild-type cells, localisation of preCOX4-mCherry (red) is equally almost completely mitochondrial, and the two markers co-localise. In respiratory-deficient ρ^0^ cells, mitochondrial import of preCOX4-mCherry (red) is no longer detected while preSU9-GFP (green) shows constitutive mitochondrial localisation; co-localisation is lost. (c) MMP based co-localisation of preSU9-GFP and preCOX4-mCherry (‘MitoLoc’), expressed as Pearson correlation coefficient (PCC). The PCC of 0.82 in exponentially growing wild-type cells is reduced to 0.55 in CCCP-treated and 0.52 in respiratory-deficient ρ^0^ cells. n = 37; * p < 0.01; error bars represent +/− SEM. (d) Wide-field microscopy images of YSBN1 wild-type cells treated with DMSO (upper panel) or 15 μM CCCP (lower panel) and imaged after the time indicated. While DMSO does not lead to a delocalisation of preCOX4-mCherry (red), CCCP treatment results in a loss of MMP and cytosolic localisation of preCOX4-mCherry; preSU9-GFP (green) remains mitochondrial. (e) Time-dependent loss of co-localisation upon treatment of yeast cells with the mitochondrial uncoupler CCCP. Wild-type YSBN1 cells were treated with 15 μM CCCP and imaged after the time indicated. Co-localisation analysis of MMP based on MitoLoc allows quantification of the gradual decline in MMP with time, eventually reaching complete delocalisation after 6 h. n = 37; * p < 0.01; error bars represent +/− SEM. (f) Wide-field microscopy images of YSBN1 wild-type cells treated with the CCCP concentration indicated and imaged after 6 h. De-localisation of preCOX4-mCherry (red) is visually evident at 15 μM CCCP, while preSU9-GFP (green) remains mitochondrial. (g) Quantification of CCCP-induced MMP loss based on co-localisation analysis of preSU9-GFP and preCOX4-mCherry by computational analysis. A pronounced loss of MMP is detectable already at 7.5 μM CCCP after 6 h of incubation. n = 35; error bars represent +/− SEM.

The method exploits mitochondrial localisation signals that are dependent on the membrane potential for protein import (Bevis and Glick, 2002; Veatch et al., 2009). And calculates the membrane potential by determining the degree of co-localisation of a second marker, that is imported in a potential-independent manner. As one of the prevailing models in metabolism research, we established the system in budding yeast allowing us a thorough examination of its functionality by testing different growth conditions, genetic mutations, and chemical treatments. For robust labelling of mitochondria independent of their MMP in yeast, we fused GFP to the fungal mitochondrial localisation signal of the F_0_-ATPase subunit 9 (preSU9) of *Neurospora crassa*, an established MMP independent localisation signal (Merz and Westermann, 2009; Schmidt et al., 1984; Westermann and Neupert, 2000). To generate the second marker, an mCherry protein was fused to the N-terminal localisation sequence of cytochrome C oxidase 4 ((COX4), preCOX4, [1:28]), which is imported into mitochondria proportional to the MMP (Bevis and Glick, 2002; Garipler et al., 2014; Veatch et al., 2009). Simultaneous expression is achieved by placing both markers on one nouresothricin-selectable centromeric plasmid, which is compatible with the majority of *Saccharomyces cerevisiae* laboratory strains (pMitoLoc, Fig. 1a, made available through Addgene).

We tested whether the degree of co-localisation between the MMP dependent and MMP independent markers could be employed as a measure of the mitochondrial potential in living cells. The co-localisation of preCOX4-mCherry and preSU9-GFP was first assessed in YSBN1 (Canelas et al., 2010), a prototrophic descendant of the widely used laboratory strain S288c. Multichannel microscopy images acquired on an Olympus IX81 wide field microscope revealed mitochondrial co-localisation of both markers, indicating import of both markers in exponentially growing wild type cells (Fig. 1b). Expressing the degree of protein co-localisation quantitatively on a pixel-by-pixel basis, this co-localisation was reflected by a correlation coefficient (PCC) of 0.82 (Fig. 1c). To experimentally reduce MMP, we then treated wild type cells with the uncoupling reagent carbonyl cyanide *m*-chlorophenyl hydrazone (CCCP), which triggers a decline in MMP due to mitochondrial membrane permeabilisation (Kasianowicz et al., 1984; Martin et al., 1991). CCCP exposure led to a gradual de-localisation of the MMP dependent preCOX4-mCherry marker, eventually reaching broad cytosolic distribution (Fig. 1d). This was reflected by a PCC minimum of 0.55, as a base level of correlation due to areas of background in both channels. The de-localisation of the marker upon uncoupling membrane potential was confirmed in both a time- (Fig. 1d,e) and dose-dependent manner (Fig. 1f,g). Hence, the degree of co-localisation detected the loss in MMP induced by the uncoupling reagent.

Next, we tested whether the method would also detect the decline of MMP in ρ^0^ cells lacking mtDNA. ρ^0^ cells cannot assemble several components of the respiratory chain, and are thus severely hampered in establishing a proton gradient (Giraud and Velours, 1997). Depletion of mtDNA led to a strong de-localisation of the MMP-dependent but not the MMP-independent marker, resulting in a PCC of 0.52 (Fig. 1b,c). This value corresponds to the effect of the highest used CCCP dose of 30 μM 6 h post treatment (Fig. 1c). Hence, co-localisation analysis using PCC also detected the decline in membrane potential caused by depletion of mitochondrial DNA. To determine the sensitivity of the analysis, we expressed the image's pixel intensities as scatter plots; facilitating expression of the loss of protein co-localisation as a reduced coefficient of determination (R^2^) for a least squares linear regression fit (Suppl. Fig. 1). A subsequent power analysis revealed that cell numbers as low as 6 are sufficient to quantify MMP using the method presented (Suppl. Fig. 2). According to these findings, measurement of MMP using a dual reporter system is feasible and reproducible, and yields a quantitative measure of mitochondrial protein import. In many cases the application of this principle is simpler than using ionic dyes for determining changes in MMP (i.e. as samples can be fixated and analyzed at a later time), and thus the method will be useful for many laboratories. In order to facilitate broad application of this principle, we wrote a freely available software plugin integrated in the ImageJ platform, which can be employed to evaluate images generated using pMitoLoc (yeast_correlation). Moreover we deposited the plasmid at Addgene (#71207) to allow access by the community.

### 2.2 Assessment of mitochondrial network volume and morphology

**Fig. 2:**
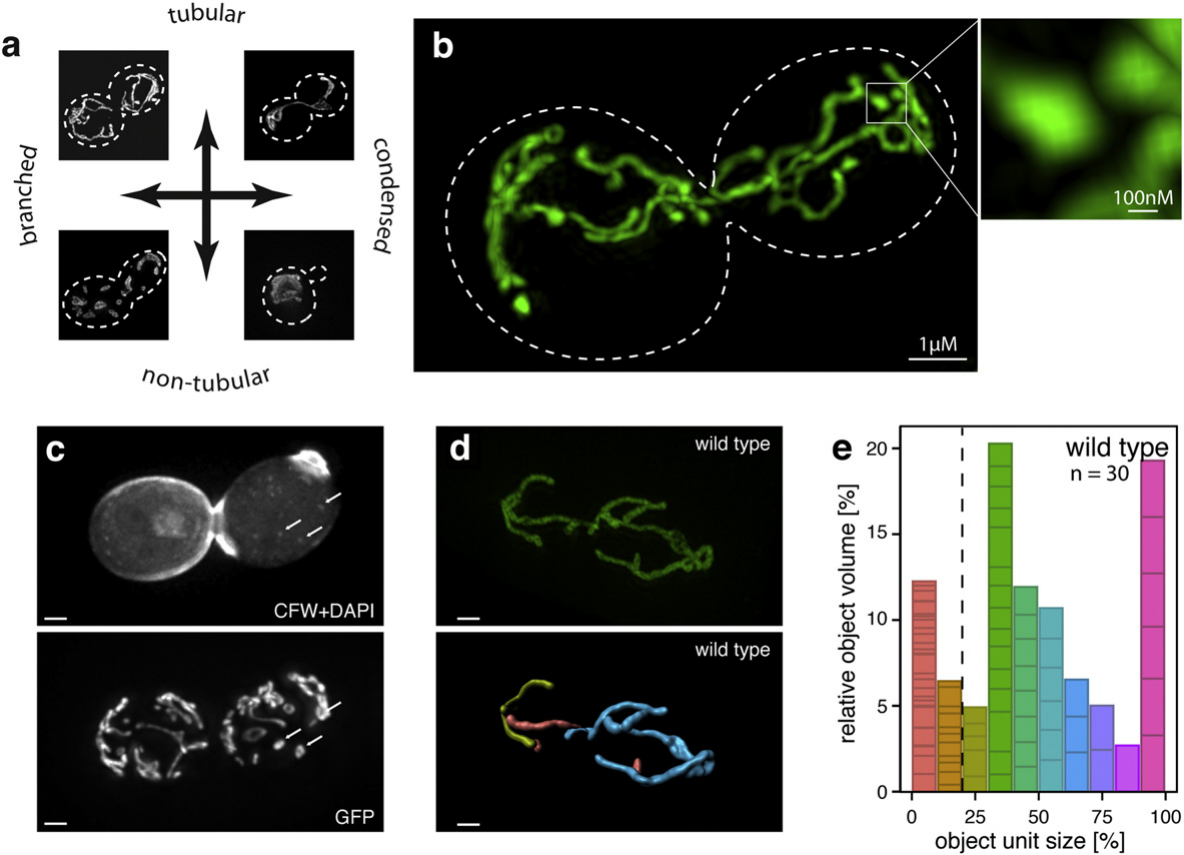
Super-resolution microscopy applied to measure network morphology of wild type yeast cells. (a) Schematic representation of mitochondrial network morphologies. While exponentially growing wild-type cells typically display a branched tubular network, oxidative damage or defects in fusion can lead to fragmentation, or condensed morphologies as a consequence of impaired mitochondrial fission. (b) 3D-SIM super-resolution microscopy applied to yeast mitochondria marked with pMitoLoc (green). Shown is the typical wild-type network morphology of an exponentially growing diploid YSBN1 cell, with resolution better than 100 nm. (c) Single fragmented mitochondrial units typically contain one mtDNA cluster. 3D-SIM super-resolution microscopy image of exponentially growing yeast cell treated with 1.0 mM H_2_O_2_, stained with DAPI (upper panel) and Calcofluor white (lower panel) to highlight DNA and the cell wall, respectively, and carrying pMitoLoc to mark the mitochondrial network. Scale bar = 1 μm. (d) Measurement of mitochondrial network morphology in wild-type cells using 3D-SIM. (Upper panel) preSU9-GFP (green) image of a typical mitochondrial network. Scale bar = 1 μm. (lower panel) 3D reconstruction of the network, with individual fragments colour-coded according to their relative size. Scale bar = 1 μm. (e) Stacked bar plot of mitochondrial fragment volumes binned by the relative contribution to the total mitochondrial volume, combined for 30 cells per genotype (population footprint). Colour coding as in (d). Objects smaller than 20% of the total volume are considered fragmented (dotted line).

Next, we used the dual reporter plasmid to measure changes in mitochondrial morphology in quantitative, numeric terms. Mitochondria respond to changes in cellular energy demand and perturbations by changing shape, form and volume. In yeast, exponentially growing wild type cells exhibit a branched, tubular morphology (Fig. 2a, top left). Upon oxidative stress, tubularity decreases as networks break up into smaller, more compact units (Fig. 2a, bottom left). Other perturbations can lead to single, condensed mitochondrial structures. We transformed pMitoLoc into YSBN1 and used structured illumination (SI) microscopy on a Deltavision 3D-SIM OMX platform to generate sub-wavelength images at ~ 120 nm resolution. The MMP-independently imported marker preSU9-GFP showed almost exclusive mitochondrial localisation (Fig. 2b) and was used for determining mitochondrial morphological features. To describe the possible changes in morphology as a function of shape, size, position and integrity, we developed 3D reconstruction and analysis software (*MitoMap*) as a plugin for ImageJ (Schneider et al., 2012). The plugin as well as the mathematical procedures and equations used to calculate the morphological parameters are given in the Materials and Methods section. For each object defined by Otsu thresholding, the software calculates two parameters for mitochondrial fragmentation (surface area and volume), two parameters for condensation (sphericity and distribution isotropy), and one parameter for tubularity (compactness).

The analysis of exponentially growing YSBN1 cells revealed that most mitochondria assemble in medium-sized units, typically forming one larger fragment which encompasses > 50% of the total mitochondrial volume within the cell (Fig. 2d, cyan). We observed that cells growing in parallel exhibit diversity in their network pattern, indicative of the cell-to-cell variability in mitochondrial network morphology (Levy et al., 2012; Rosenfeld et al., 2005). For instance, six out of 30 wild-type cells possessed a single mitochondrial network comprising more than 90% of the total mitochondrial volume. This is visualised in a superimposed colour-coded histogram (Fig. 2e), where magenta represents units with 90–100% of the total volume. Around one fifth of mitochondrial network units were smaller than 20% of the total volume. Co-staining with DAPI indicated that these small units typically contained no more than one mitochondrial DNA cluster, indicating that they represent ‘single’ mitochondria (Fig. 2c). Based on this information, we introduced a simple fragmentation index *f* indicative of the relative occurrence of the small units in relation to the volume of the fused network. This value allows a quantitative comparison of mitochondrial fragmentation between different genotypes or conditions, and across different experiments. The fragmentation index *f* is defined as the sum of relative fragment volumes that individually constitute less than 20% of the total mitochondrial volume. Wild-type YSBN1 cells in SC media, calculated from 30 cells each to obtain a population estimate, reproducibly resulted in f = 20.7 ± 3.3.

### 2.3 Differentiating mitochondrial fission phenotypes on the basis of *MitoMap* geometrical classifiers

**Fig. 3:**
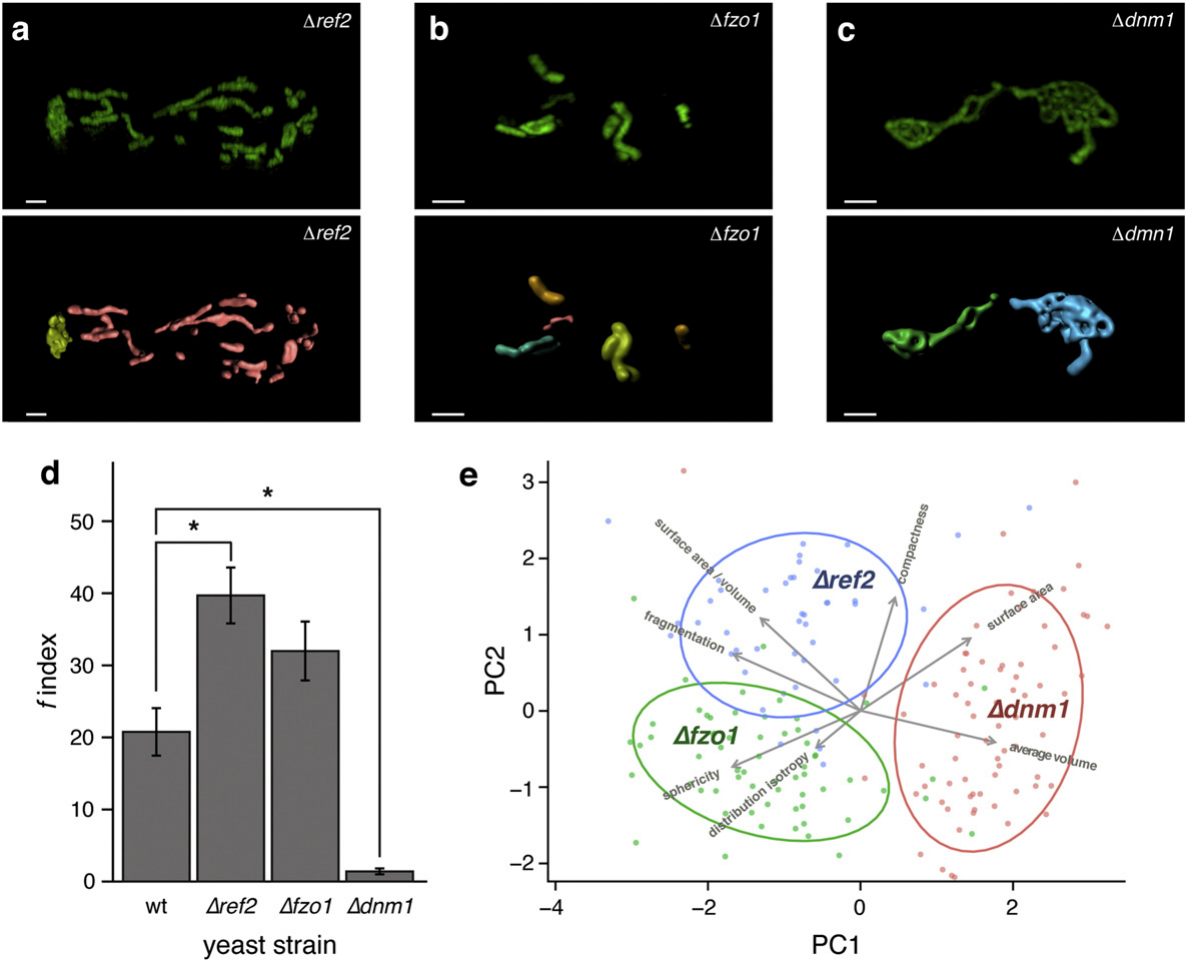
Quantitative analysis of mitochondrial network morphologies in fusion/fission protein deletion mutant cells. (a–c) Measurement of mitochondrial network morphology in yeast cells with defects in fusion/fission proteins using 3D-SIM. (upper panel) PreSU9-GFP (green) image of a typical mitochondrial network in a) ∆*ref2*, b) ∆*fzo1* and c) ∆*dnm1* cells. Scale bar = 1 μm. (lower panel) 3D reconstruction of the network, with individual fragments colour-coded according to their relative size. Scale bar = 1 μm. Images are directly comparable to wild-type cells shown in Fig. 1d. (d) Quantification of the fragmentation index *f* of mitochondrial networks in wild type and single-gene deletion mutants. n = 30; * p < 0.01; error bars represent +/− SEM. (e) Principal component (PC) analysis of mitochondrial geometry determined by preSU9-GFP and super-resolution microscopy. Separation in PC1 is mostly driven by fragmentation, while sphericity and compactness contribute to separation in PC2. n = 30 cells per genotype.

We applied *MitoMap* to evaluate the mitochondrial network in yeast cells deleted for genes important for mitochondrial fission and fusion. ∆*ref2* (Ferrer-Dalmau et al., 2010) and ∆*fzo1* (Rapaport et al., 1998) cells are reported to possess a fragmented mitochondrial network (Dimmer et al., 2002). Analysis with our reporter system confirmed these results, and allowed us to express the changes in quantitative terms. In contrast to wild-type cells (Fig. 3d), ∆*ref2* and ∆*fzo1* mutants did not contain any large mitochondrial network units at all. Instead, their mitochondrial structures consisted of mostly small and medium-sized fragments resulting in fragmentation indexes of 39.7 ± 3.8 and 32.0 ± 4.1, respectively (Fig. 3a,b). In contrast, small mitochondrial units were no longer observed upon deletion of dynamin 1 (∆*dnm1*), a gene required for mitochondrial fission (Bleazard et al., 1999). Here, the mitochondria in most cells assembled into a single tubular network (f = 1.4 ± 0.4, Fig. 3c).

While the simple fragmentation index distinguished the mutants from the wild-type strain, the single parameter was not sufficient to discriminate the mutant phenotypes. For instance, despite different mechanisms that lead to network fragmentation in ∆*ref2* (Hermann et al., 1998) and ∆*fzo1* (Ferrer-Dalmau et al., 2010) cells, *f* is not significantly different in the two strains (Fig. 3a,b). These phenotypes were however differentiated by the shape descriptors calculated by the *MitoMap* plugin, which estimates tubularity and condensation by calculating sphericity, distribution isotropy and compactness using the equations detailed in Material and Methods. While sphericity is maximal for a sphere and decreases for example with elongation along one axis, distribution isotropy is more complex. Here, the sum of the ratios of second moments for each combination of the three principal axes, x:y, x:z and y:z are taken into account, giving minimal values for rotationally symmetric objects. The value increases for example in objects that are compact in one orientation and diffuse in another. Finally, compactness estimates how regularly shaped an object is by dividing radius variance by volume. As small fragments *per se* are inherently more compact and spherical, we corrected for this by weighting each object's features using their surface area before calculating the mean value for each feature and cell.

When combined, these morphological classifiers allow expression of and discrimination between subtle changes in mitochondrial morphology based on objective, numeric criteria. In a principal component analysis (PCA), six of the calculated features plus single-cell level fragmentation index separated ∆*ref2*, ∆*fzo1* cells into three groups, which fully correspond to their genotype (Fig. 3e). The main separation along the first component was driven by fragmentation, with the two heavily fragmented strains ∆*ref2* and ∆*fzo1* separating from the condensed strain ∆*dnm1*. Separation along the second component was driven by sphericity and compactness, allowing clear distinction of the mitochondrial network morphologies of ∆*ref2* and ∆*fzo1* cells (Fig. 3e). Thus, *MitoMap* reports quantitative morphological parameters that distinguish the mitochondrial networks of ∆*ref2* and ∆*fzo1* cells, despite their similar fragmentation pattern.

### 2.4 The correlation of mitochondrial membrane potential and fragmentation in fission/fusion mutants and ρ^0^ cells

**Fig. 4:**
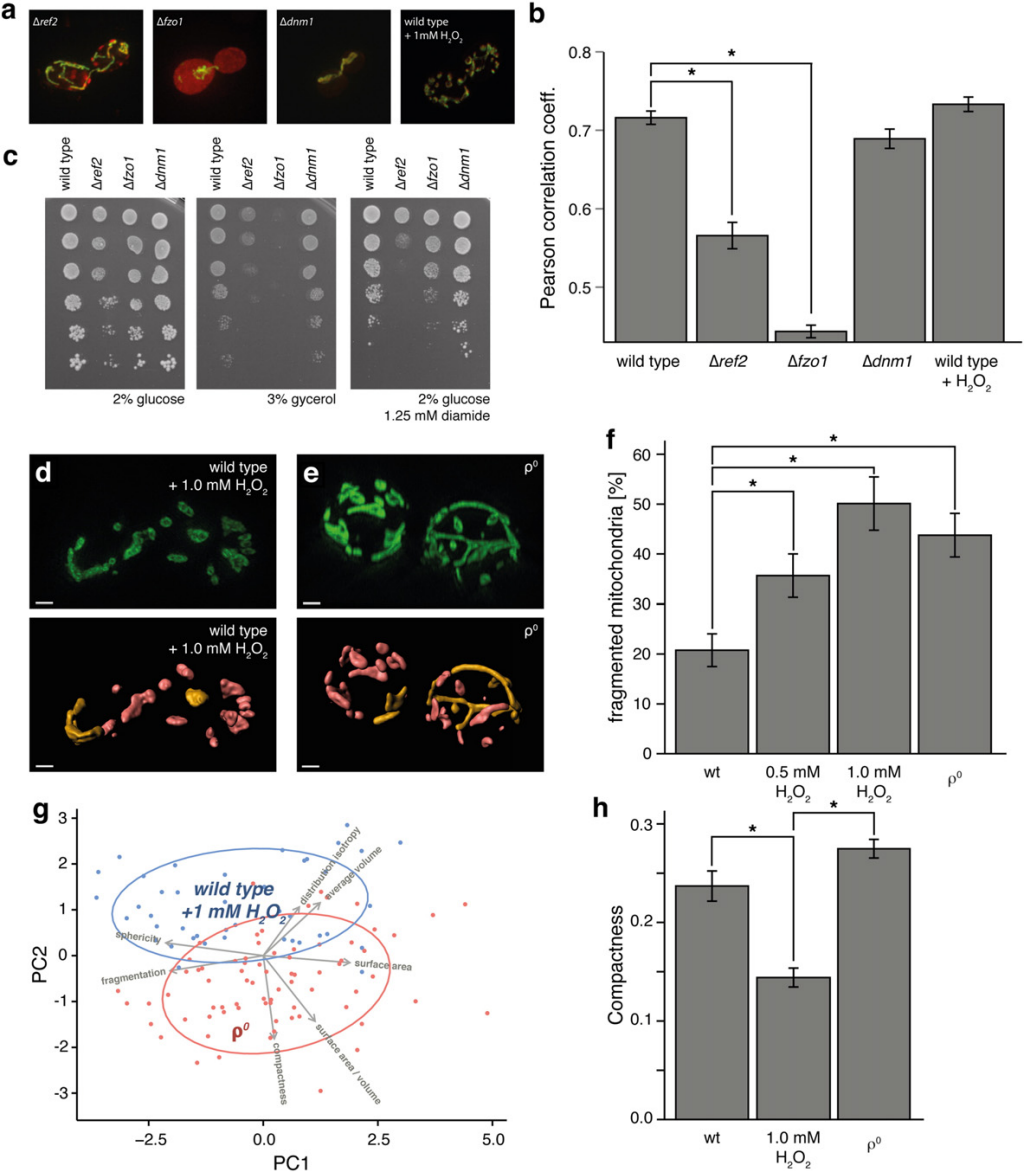
MitoLoc employed for analysis of the relationship between mitochondrial membrane potential and morphology. (a) Wide-field microscopy images of BY4741 yeast cells with deletions of mitochondrial fusion/fission factors *REF2*, *FZO1* and *DNM1*, or treated with 1 mM H_2_O_2_ for 45 min. Green, preSU9-GFP; red, preCOX4-mCherry. (b) Quantification of the mitochondrial membrane potential based on co-localisation analysis. ∆*ref2* and ∆*fzo1* cells show reduced membrane potential. n > 87; * p < 0.01; error bars represent +/− SEM. (c) Growth on the non-fermentable carbon source glycerol (middle panel) is impaired in ∆*ref2* and ∆*fzo1* strains, but unaffected in ∆*dnm1* yeast. Magnitude of the growth defect is correlated with MMP loss. (right panel) Resistance against 1.25 mM diamide is reduced in ∆*ref2* and ∆*fzo1*, but comparable to wild type in ∆*dnm1*. (d,e) Mitochondrial network morphology in wild-type cells treated with 1 mM H_2_O_2_ for 45 min and respiratory-deficient ρ^0^ determined using 3D-SIM. (upper panels) PreSU9-GFP (green) image of a typical mitochondrial network. Scale bar = 1 μm. (lower panels) 3D reconstruction of the network, with individual fragments colour-coded according to their relative size. Scale bar = 1 μm. (f) Quantification of the fragmentation grade in mitochondrial networks of wild type cells treated with 0.5 or 1 mM H_2_O_2_ for 45 min and respiration-deficient ρ^0^ cells. n = 30; * p < 0.01; error bars +/− SEM. (g) Principal component analysis separates mitochondrial geometry of H_2_O_2_ treated and respiratory-deficient ρ^0^ cells as determined by preSU9-GFP and super-resolution microscopy. Separation in PC1 is mostly driven by fragmentation, while sphericity and compactness contribute to separation in PC2. n = 30 cells per genotype. (h) The *MitoMap* parameter representing tubularity (compactness) determined in wild type yeast cells, cells treated with 1 mM H_2_O_2_ for 45 min and respiratory-deficient ρ^0^ cells. n > 42; * p < 0.01; error bars represent +/− SEM.

We combined both aspects of the new method to illustrate the extent to which mitochondrial fission/fusion and changes in the MMP correlate. First, we compared morphological parameters and MMP in deletion mutants of the mitochondrial morphology factors *DNM1*, *REF2* and *FZO1*. Deletion of *DNM1*, preventing mitochondrial fission and resulting in large tubular network structures, did not affect the MMP. The deletion of *REF2* and *FZO1*, however, triggered a significant loss of MMP (Fig. 4a,b). This corresponded to the inability of the mitochondria to utilise non-fermentable carbon sources such as glycerol, and correlated with increased cellular sensitivity to the oxidative stressor diamide (Kosower et al., 1969) (Fig. 4c). The decline in MMP detected by pMitoLoc/*MitoMap* thus predicted a respiratory deficiency in the ∆*fzo1* strain that was confirmed by growth analysis (Fig. 4c). Remarkably, the moderate loss of MMP identified in ∆*ref2* was also reflected in a partial growth inhibition on glycerol, implying a high sensitivity of the assay.

H_2_O_2_ treatment has been reported to induce mitochondrial network fission (Wu et al., 2011) but, at least at moderate levels, to leave the MMP intact (Bajić et al., 2013; Konno and Kako, 1991). We tested whether this phenotype would be confirmed with our method. Treatment of pMitoLoc-carrying yeast cells with 1.0 mM H_2_O_2_ for 45 min resulted in a pronounced fission of the mitochondrial network (f = 50.1 ± 5.3) (Fig. 4b,d,f). The MMP was however not affected by this degree of H_2_O_2_ treatment, and remained at wild-type levels (Fig. 4b). This result was confirmed by staining with the cationic dye DiOC_6_ (Suppl. Fig. 3).

In yeast cells, loss of a detectable MMP has been described to co-occur with fragmentation of mitochondrial structures (Aerts et al., 2009). The pMitoLoc/*MitoMap* method confirmed the mitochondrial network of ρ^0^ cells to be highly fragmented (f = 43.7 ± 4.3), and we observed a strong loss of MMP (Fig. 4e,f). Despite the fact that this fragmentation might arise from the higher ROS load observed in ρ^0^ cells (Grant et al., 1997; Indo et al., 2007), the *MitoMap* pipeline clearly distinguished the mitochondrial networks of H_2_O_2_ treated from that of ρ^0^ cells, despite their fragmentation index being the same. In a PCA, the two conditions are separated mainly along the second component, driven by compactness and distribution isotropy (Fig. 4g). When evaluating compactness alone, ρ^0^ mitochondria show a modest increase in the compactness value compared to wild type mitochondria, while wild type cells treated with 1.0 mM H_2_O_2_ present with a highly significant reduction in compactness (Fig. 4h). In other words, the heavy fragmentation of mitochondrial structures resulting from H_2_O_2_ treatment produces near spherical objects with high symmetry. In contrast, fragmentation arising from the lack of mitochondrial DNA, resulting in the absence of essential components of the electron transport chain produces elongated structures of various symmetries.

## 3 Discussion

Despite the importance of dynamic rearrangements (fission and fusion) of mitochondrial networks and their influence on cellular physiology, ageing and metabolic disease being universally accepted (Karbowski and Youle, 2003; Shaw and Nunnari, 2002), mitochondrial morphologies are still reported in the vast majority of current scientific literature as arbitrary, non-quantitative interpretation of microscopical images. This makes comparison of published literature subjective, and thus many published research findings cannot be directly compared to each other. In the present manuscript, we address this bottleneck and propose an easy to implement workflow that facilitates the numerical description of both network morphology and membrane potential in mitochondria of single cells.

The method is based on differential mitochondrial import of two florescent protein markers, one of which (preSu9-GFP) is constitutively imported into mitochondria and used to mark mitochondrial structures, while a second marker (preCox4-mCherry) is imported dependent on the mitochondrial inner membrane proton gradient, and used to quantify the mitochondrial membrane potential on the basis of pixel-by-pixel co-localisation analysis. We show that this approach can be used to quantify MMP by measuring the decrease induced by treatment with the uncoupler CCCP, where both a time-dependent and a dose-dependent effect can be observed. We also evaluate the concept in yeast cells depleted of mtDNA, where the absence of a functional MMP has been described (Giraud and Velours, 1997; Veatch et al., 2009).

The workflow presented can be used as an alternative method to measure mitochondrial membrane potential which can complement established methods that rely on cationic dyes (DiOC_6_, Rhodamine-123, JC-1). Dye-based approaches to MMP measurement are problematic, for instance when cellular ion import is impaired as it might be the case in ρ^0^ cells. In addition, most MMP-sensitive dyes are not fixable, and require careful synchronisation of cell treatment and microscopy in order not to introduce artefacts. In contrast, fixation of the fluorescent proteins constituting the proposed method preserves their localisation and allows image acquisition even days after the actual experiment.

While functional in all tested examples, it has to be noted that certain limits apply to this method. Most importantly, changes in MMP can happen on a timescale of a few seconds (Perry et al., 2011), while localisation loss of preCOX4-mCherry is slower due to the kinetics of protein transport. The MitoLoc method presented here is thus not suitable for monitoring fast kinetics in the change of MMP. Another limit is that the result of an MMP measurement can be influenced by cofounding effects that affect membrane transport independent of the MMP. We cannot exclude such effects for all possible experiments and recommend therefore that, as with every novel method, unexpected new findings should be supported with at least one independent technology.

Although the images used in this paper have been acquired on high-end microscopes not available in every laboratory, application of the MitoLoc method is not restricted to these. To evaluate this, we measured MMP on a conventional fluorescence microscope. While measurement of only 6 cells was sufficient to obtain a statistically significant discrimination of CCCP-treated and wild type cells on a Deltavision Olympus IX81 microscope with image deconvolution (Suppl. Fig. 2), we were also able to measure a highly significant (p < 10^− 7^) difference between CCCP-treated and control cells when analysing 50 cells per condition using a very basic, manually operated Olympus BX51 fluorescence microscope (data not shown). An even simpler approach would be to express the markers from pMitoLoc and to count the number of cells with apparent delocalisation of the MMP-dependent marker. Despite being less sensitive, similar approaches were sufficient in to detect MMP changes in previous studies (Garipler et al., 2014; Veatch et al., 2009).

The main advantage when using the method on advanced microscopes is certainly that it allows, in parallel with measurement of the membrane potential, enumeration of mitochondrial morphological parameters. To simplify such analysis for day-to-day laboratory use, we developed and implemented a software plugin that contains an advanced quantitative analysis pipeline for measurement of mitochondrial morphologies. Here, only the constitutively imported GFP marker (preSu9-GFP) is imaged using super-resolution fluorescence microscopy. The software uses 3D reconstruction algorithms to calculate volume, surface area and the more sophisticated shape descriptors radius variance, compactness, distribution isotropy, isoperimetric quotient and sphericity for every object in a given cell separately. We show the usefulness of these quantitative parameters by evaluating the mitochondrial morphologies of wild-type cells, fission/fusion mutants, ρ^0^ cells and oxidant-exposed yeast. Using our pipeline, we found that deletion of *REF2* and *FZO1* lead to a profound fragmentation of mitochondrial networks with up to 40% of the mitochondrial volume contained in small fragments, and confirmed that deletion of *DNM1* results in a heavily condensed network with no small fragments present. Although the computational reconstruction is not required to detect mitochondrial fission *per se* (easily detected by visual inspection), it is required for the differentiation of complex morphologies such those caused by Δ*ref2* and Δ*fzo1* deletion, or distinguishing the fragmentation upon H_2_O_2_ treatment or upon depletion of mitochondrial DNA, which are characterised by a similar degrees of fragmentation. Our workflow was able to separate these genotypes based on the calculated mitochondrial morphological parameters, reflecting the different mechanisms leading to fragmentation.

As mitochondrial morphologies vary between branched and condensed as well as tubular and non-tubular networks in a quite dynamic way, we have included a variety of shape descriptors suitable for many conditions in the ImageJ plugin *MitoMap*. It should be noted that not all descriptors are useful in all circumstances, and the ones best representing the factors of interest of each experiment should be chosen for comparison. We provide the dual marker construct (pMitoLoc) (deposited at Addgene, #71207 (http://www.addgene.org)) that is compatible with the majority of laboratory *S. cerevisiae* strains, and release our software as open-source plugins for ImageJ (accessible at http://www.gurdon.cam.ac.uk/stafflinks/downloadspublic/imaging-plugins). Although geometrical analysis of cellular structures is certainly possible with other algorithms and software, this plugin is specifically written to estimate the parameters required to describe mitochondrial network morphology and membrane potential, is easy to use, open source, and thus ideal for academic research on mitochondrial networks. We established the system in budding yeast, however the software is applicable in other organisms with the only requirements being the possibility to transfect cells and the existence of mitochondrial-localised florescent protein markers, which are available now for many organisms (Baqri et al., 2009; Logan and Leaver, 2000; Rizzuto et al., 1998). Notably, the software allows re-analysis of previously acquired images, and may thus be applied to quantify mitochondrial morphology in existing data.

In summary, we present a novel method for the quantification of mitochondrial membrane potential, and an easily applicable workflow for the reliable and parallel quantification of mitochondrial network morphology functional in single cells. Applied to yeast, we elaborate changes in mitochondrial membrane potential and morphology induced by genetic deficiency in fission and fusion, under oxidative stress, in cells lacking mitochondrial DNA, and in uncoupled cells. We propose that employing numerical classifiers for the reporting of mitochondrial morphological parameters increases the value and reproducibility of mitochondrial morphology description.

## 4 Methods

### 4.1 Yeast strains, media and plasmids

All experiments involving wild-type yeast strains were carried out using YSBN1, a prototrophic diploid variant of *S. cerevisiae* S288c (Canelas et al., 2010). Yeast strains deleted for proteins involved in mitochondrial morphology were obtained from the yeast gene deletion collection (Winzeler et al., 1999). Cells were cultured in YPD medium (1% yeast extract, 2% peptone, 2% glucose) or synthetic complete (SC) medium with 100 μg/mL Nourseothricin (Werner BioAgents) for a minimum of 4 h to ensure log-phase growth. Where indicated, cells were treated with 1 mM H_2_O_2_ (Sigma) 45 min prior to collection, or with CCCP (Sigma) at the time and concentration indicated. Yeast cells depleted of mtDNA (ρ^0^) were generated by plating onto YPD agar containing 0.1 g/L ethidium bromide and incubation at 30 °C for 2 d. Absence of mtDNA was confirmed in isolated surviving clones by DAPI staining and growth assays using the non-fermentable carbon sources ethanol and glycerol. Staining with DiOC_6_ (Invitrogen) was performed according to manufacturer's instructions, and cells were mounted on agarose pads containing the respective culture conditions.

pMitoLoc was constructed by first replacing the *URA3* marker gene of pUG35 (Niedenthal et al., 1996) with the nouresothricin marker (NAT) from pAG25 using homologous recombination cloning. Then, the preSU9 localisation sequence of pYES_mtGFP (a gift from B. Westermann (Westermann and Neupert, 2000)) was inserted 5′ of the yEGFP gene using *Bam*HI and *Eco*RV sites. Subsequently, we used the plasmid's *Sac*I and *Bst*BI sites to introduce the preCOX4-mCherry gene of pHS12-mCherry (a gift from C. Dunn (Sesaki and Jensen, 1999)), resulting in a dual-reporter *CEN6* plasmid we termed pMitoLoc. The plasmid is made available through Addgene (www.addgene.org), plasmid ID #71207.

### 4.2 Fluorescence microscopy

For microscopy, ~ 6 × 10^6^ yeast cells were collected by centrifugation, washed twice in PBS and re-suspended in formaldehyde solution (4 g/L PFA, 3.6% sucrose) to preserve mitochondrial morphology. After 15 min, cells were washed in PBS, and where indicated 2.5 μg/mL DAPI or 5 μg/mL Calcofluor White (Sigma) was added. After one more washing step with PBS, cells were resuspended in 20 μL Vectashield mounting medium (Vector Labs). 2 μL of this mixture was applied to poly-l-lysine coated microscope slides. For live-cell microscopy, cells were mounted on agarose pads.

Super-resolution fluorescence microscopy for morphological analysis was carried out using a Deltavision 3D-SIM OMX system (GE Healthcare) equipped with a 100 × 1.4NA oil objective (Olympus), 405 nm, 488 nm and 594 nm laser lines, and the OMX Standard filter set drawer.

Images were acquired in structured illumination mode using a Z-spacing of 125 nm, and reconstructed using Softworx software (GE Healthcare). Conventional widefield fluorescence microscopy was carried out using an Olympus IX81 wide field microscope (Deltavision, GE Healthcare) equipped with a 60 × 1.42NA PlanApoN oil objective (Olympus) and an LED light source capable of delivering 405 nm, 488 nm and 594 nm excitation wavelengths. The filter sets used were FITC (490/20ex 528/38em), TRITC (555/28ex 617/73em) and DAPI (360/40ex 457/50em), and images with a Z-spacing of 200 nm were recorded with a CoolSNAP HQ2 CCD camera. Deconvolution was performed using Softworx software. For standard fluorescence microscopy, cells were examined under an Olympus BX51 microscope using filters YGFP (GFP) and HcRed1 (mCherry). Images were recorded with the help of QImage software.

### 4.3 Mitochondrial morphology analysis

Super-resolution images were analysed by the software plugin Yeast_MitoMap (available *via* web supplement and from http://www.gurdon.cam.ac.uk/stafflinks/downloadspublic/imaging-plugins) in ImageJ (Schneider et al., 2012). *MitoMap* automates the process of defining GFP-labelled mitochondria in a selected region of interest and calculates their volume, surface area and shape descriptors using the formulae listed in Table 1. Documentation on how to use the plugin is included in the supplementary material. 32-bit OMX image stacks are converted to 16-bit and Otsu thresholding (Otsu, 1979) is used to extract the labelled volume. From this volume, surface voxels are defined as those having at least one exposed face and assigned to the classes defined by Mullikin and Verbeek (Mullikin and Verbeek, 1993) extended with additional classes to allow for different dimensions in xy and z. This gives a total of 15 different possible surface voxel configurations, each with a weighting factor used to estimate their contribution to the 3D object surface area. This method was validated by comparing the estimated surface areas of binary voxel representations of spheres to the calculated volumes of spheres with the same radii.

For each single or dividing cell, a ROI was chosen that contained no other cells. Generally, cells with the highest absolute intensities were chosen where more than 30 cells had been acquired. To exclude artefacts, objects with a volume smaller than 0.1 μm^3^ were excluded.

For geometric analysis, each object's features were weighted by the respective surface area to minimize over-representation of small objects. For fragmentation analysis, relative volumes V_s_ of each cell's objects were calculated. Then, relative volumes from 30 cells per genotype were added using a V_s_ binning of 10 considering objects with V_s_ ≤ 20 as fragmented (Table 1). In the case of H_2_O_2_-treated yeast, we observed a heterogeneous population of ~ 40% cells with wild type mitochondria, while the remaining cells presented with a heavily fragmented mitochondrial network. This observation was reproducible and occurred in spite of vigorous mixing after H_2_O_2_ addition. We therefore excluded non-responding cells from the analysis.

Data was plotted using R ‘ggplot2’ and ‘ggbiplot’ packages. Imaris software (Oxford Instruments) was used to generate 3D renderings of yeast mitochondria.

### 4.4 Mitochondrial protein import analysis

Colocalisation of preSU9-GFP and preCOX4-mCherry was quantified using images acquired with conventional resolution by first cropping each image to contain one single or dividing yeast cell. Cropping was based on Calcofluor White staining in order to avoid artefacts. Image stacks were subjected to colocalisation analysis in Volocity software (Perkin Elmer) without defining further ROIs, as automatic cell shape definition using brightfield images or Calcofluor staining proved unreliable.

Alternatively, image stacks were analysed using ImageJ plugin yeast_correlation (available *via* web supplement and from http://www.gurdon.cam.ac.uk/stafflinks/downloadspublic/imaging-plugins), where cell areas for PCC analysis were defined by applying the Otsu thresholding method (Otsu, 1979) to images convolved with a Gaussian blur (σ = 5).

### 4.5 Oxidant tolerance tests

Exponentially growing yeast cells were collected and spotted in 1/5 serial dilutions onto SC (6.8 g/L YNB (Sigma), 0.59 g/L CSM (MP Biomedicals)) containing 2% glucose and 1.25 mM diamide (Sigma), or 3% glycerol where indicated. Growth was documented after 3 days incubation at 30 °C.

## Figures and Tables

**Table 1:** Mathematical formulae used to describe mitochondrial morphology.

Feature	Description and formula
Compactness	Variance of radial distance/volume
n = number of voxels making up the object; p(k) = object voxel k; d(k) = distance of object voxel k from the object centroid; V = object volume $\begin{array}{l} d\left(k\right)=\sqrt{{\left(c_{x}-p{\left(k\right)}_{x}\right)}^{2}+{\left(c_{y}-p{\left(k\right)}_{y}\right)}^{2}+{\left(c_{z}-p{\left(k\right)}_{z}\right)}^{2}}\\ \;\mathrm{Compactness}=\frac{\left(\frac{{\sum_{k=0}^{n}\left(d\left(k\right)-\bar{d}\right)}^{2}}{n}\right)}{V} \end{array}$
Distribution isotropy	The sum of ratios of the second moments in each combination of orientations
$\begin{array}{l} d{\left(k\right)}_{x}=\sqrt{{\left(c_{y}-p{\left(k\right)}_{y}\right)}^{2}+{\left(c_{z}-p{\left(k\right)}_{z}\right)}^{2}}\\ d{\left(k\right)}_{y}=\sqrt{{\left(c_{x}-p{\left(k\right)}_{x}\right)}^{2}+{\left(c_{z}-p{\left(k\right)}_{z}\right)}^{2}}\\ d{\left(k\right)}_{z}=\sqrt{{\left(c_{y}-p{\left(k\right)}_{y}\right)}^{2}+{\left(c_{x}-p{\left(k\right)}_{x}\right)}^{2}} \end{array}$ $\mathrm{Isotropy}=\frac{\left(\frac{\sum_{k=0}^{n}{\left(d{\left(k\right)}_{x}-{\bar{d}}_{x}\right)}^{2}}{n}\right)}{\left(\frac{\sum_{k=0}^{n}{\left(d{\left(k\right)}_{y}-{\bar{d}}_{x}\right)}^{2}}{n}\right)}+\frac{\left(\frac{\sum_{k=0}^{n}{\left(d{\left(k\right)}_{x}-{\bar{d}}_{x}\right)}^{2}}{n}\right)}{\left(\frac{\sum_{k=0}^{n}{\left(d{\left(k\right)}_{z}-{\bar{d}}_{x}\right)}^{2}}{n}\right)}+\left(\frac{\frac{\sum_{k=0}^{n}{\left(d{\left(k\right)}_{y}-{\bar{d}}_{y}\right)}^{2}}{n}}{\frac{\sum_{k=0}^{n}{\left(d{\left(k\right)}_{z}-{\bar{d}}_{z}\right)}^{2}}{n}}\right)$
Isoperimetric quotient	The ratio of the object volume to the volume of a sphere with the same surface area
A = object surface area $IPQ=\frac{V}{\frac{4}{3}\pi {\left(\frac{A}{4\pi }\right)}^{3}}$
Sphericity	The ratio of the surface area of a sphere with the same volume as the object to the surface area of the object
$\mathrm{Sphericity}=\frac{\pi^{\frac{1}{3}}6V^{\frac{2}{3}}}{A}$
Radius variance	A measure of how smooth the radial distance to the surface is, 0 for a sphere
r(j) = distance of object surface voxel j from the object centroid $\begin{array}{l} r\left(j\right)=\sqrt{{\left(c_{x}-p{\left(j\right)}_{x}\right)}^{2}+{\left(c_{y}-p{\left(j\right)}_{y}\right)}^{2}+{\left(c_{z}-p{\left(j\right)}_{z}\right)}^{2}}\\ \mathrm{Radius}\;\mathrm{variance}\;=\frac{\sum_{j=0}^{n}{\left(r\left(j\right)-\bar{r}\right)}^{2}}{n} \end{array}$
Fragmentation	$V_{s}=\frac{V_{\mathrm{fragment}}}{V_{\mathrm{total}}}\times 100$ $f=\frac{\sum_{1}^{30}V_{s\leq 20}}{\sum_{1}^{30}V_{s}}$

## Appendix A Supplementary data

Supplementary material: ImageJ plugins.
