# Supplementary material for: MitoLoc: A method for the simultaneous quantification of mitochondrial network morphology and membrane potential in single cells
Source: Mitochondrion. 2015 Sep;24:77–86. doi: 10.1016/j.mito.2015.07.001 (PMC4570932; doi:10.1016/j.mito.2015.07.001)
Supplement: Supplementary file 1 — Supplementary material: ImageJ plugins. [file mmc1.zip › MitoLoc_plugins/readme.pdf]

Yeast MitoMap

Vowinckel et al., 2015 - http://www.ncbi.nlm.nih.gov/pubmed/26184437

Introduction

This plugin automates the process of defining GFP-labelled mitochondria in a selected region of interest and calculates various shape descriptors that represent the overall distribution of signal in different ways.

Installing and Running the Plugin

When installed by placing Yeast_MitoMap.jar in the Fiji plugins folder, a menu item to run the plugin will be added to the Analyse menu. The GUI is very simple, giving the option to visualise the mapped volume in 3D Viewer [1] and providing an "Analyse" button to run the analysis on the current image stack. A selection is required to define the XY area of the image stack to be analysed.

Pipeline Overview

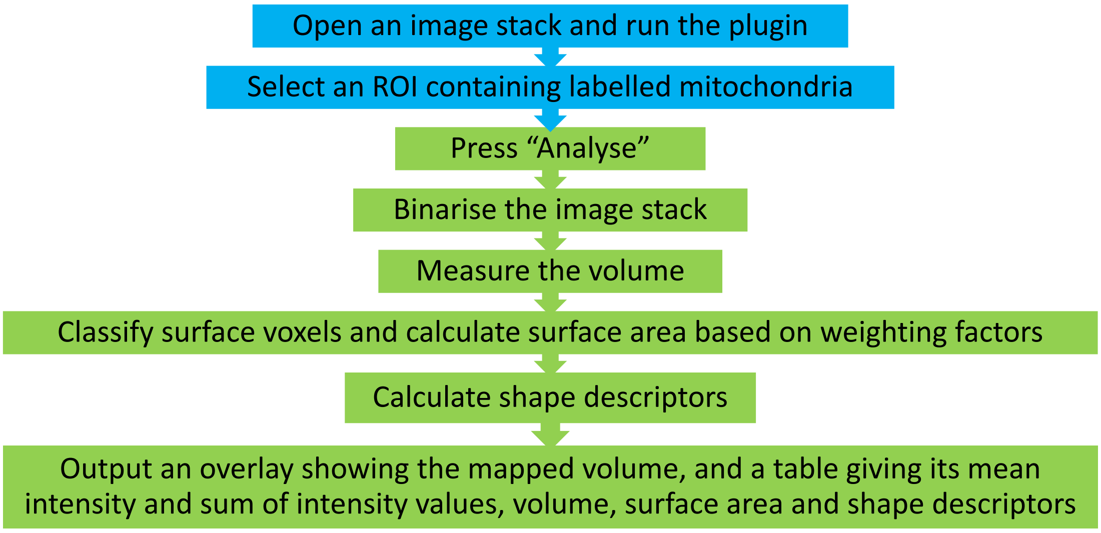

Yeast MitoMap is easy to use, the automated pipeline requires only selection of the volume to analyse before the processing and analysis is run without user input.

Signal Mapping

Otsu thresholding [2] is used to extract the labelled volume, and from this volume surface voxels are defined as those having at least one exposed face and assigned to the classes defined by Mullikin and Verbeek [3] extended with additional classes to allow for different dimensions in xy and z. This gives a total of 15 different possible surface voxel configurations, each with weighting factors used to estimate their contribution to the 3D object surface area. Voxels are discrete sampled points in image space taken from a continuous system in object space, which makes their computational representation as cuboids is useful but potentially misleading. The weighting factors were calculated by Mullikin and Verbeek to estimate the area represented by the voxel configurations given that they are points on a curved surface, and assuming that the sampling rate is sufficient so that the surface is as flat as possible between connected voxels. While exact recovery of the sampled surface is not possible, the local estimation errors largely cancel out, as shown by the results from voxel renderings of spheres which have errors of less than 2%.

Class	Configuration	Horizontal Faces	Vertical Faces	Estimated Surface Area (H = voxel width/height, V = voxel depth)
1	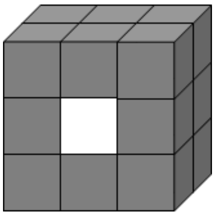	0	1	$0.894\mathrm{HV}$
2	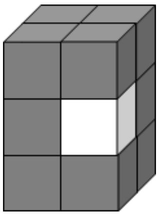	0	2	$1.3409\mathrm{HV}$
3	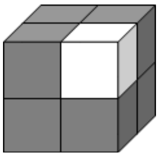	1	2	$2\mathrm{HV} + 1.5879\frac{\mathrm{HV}}{3}$
4	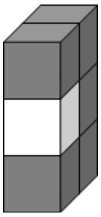	0	3	$2\mathrm{HV}$
5	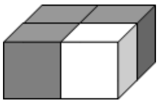	2	2	$2\mathrm{HV} + \frac{2HV}{4}\frac{8}{3}$
6	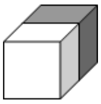	2	3	$3\mathrm{HV} + \frac{2HV}{3}\frac{10}{3}$
7	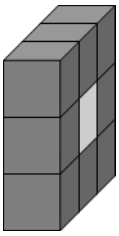	0	2	$2\mathrm{HV}$
8	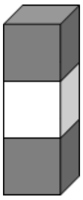	0	4	$4\mathrm{HV}$
9	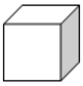	2	4	Not included
10	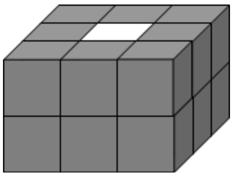	1	0	HV

11	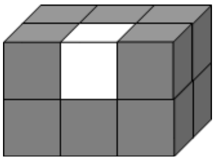	1	1	$\mathrm{HV} + 1.3409\frac{HV}{2}$
12	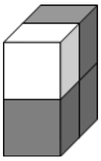	1	3	$3\mathrm{HV} + \frac{HV}{4}\frac{8}{3}$
13	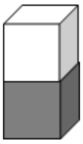	1	4	$4\mathrm{HV} + \frac{HV}{3}\frac{10}{3}$
14	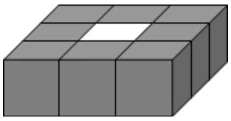	2	0	$2\mathrm{HV}$
15	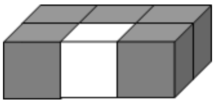	2	1	$\mathrm{HV} + 1.5879\frac{2HV}{3}$

The voxel classes and formulae used to estimate surface area. Classes 1-9 were defined by Mullikin and Verbeek [3], classes 10-15 use the same weight values to allow different calibrations in XY and Z.

Sphere Radius	$4\pi r^2$	Yeast MitoMap Estimation	Error	% Error
10	1256.637	1275.673	19.0359	1.5148
12	1809.557	1773.743	35.8144	1.9792
14	2463.009	2440.825	22.1836	0.90067
16	3216.991	3180.471	36.5199	1.1352
18	4071.504	4068.893	2.6111	0.064131
20	5026.548	5028.927	2.3788	0.047325

Validation of the surface area estimation algorithm by comparing calculated surface areas of spheres to the Yeast MitoMap estimation of their surface area when rendered as voxels.

Shape Descriptors

These values are calculated to give different representations of the distribution of signal in the volume of interest that are comparable between samples. It is likely that not all of these values will be required for a given experiment, and the ones best representing the factors of interest should be chosen for comparison.

Surface area:volume is the simplest shape descriptor, and is a measure of size and compactness.

Radius variance is the variance of the distance from the object centroid to the closest point on the surface divided by the object volume, giving 0 for a sphere and higher values as the shape becomes less regular, independent of how closely packed it is.

Compactness is the radius variance divided by the object volume. This gives 0 for a sphere and higher values for signal that is either less regular or more diffuse.

Distribution isotropy is the sum of the ratios of second moments for each combination of the three principal axes, x:y, x:z and y:z. The second moment is the variance with respect to a single axis, so the smallest values are given by objects that are rotationally symmetric about all three axes, and the largest represent objects that are compact in some orientations and diffuse in others.

The **isoperimetric quotient** is the ratio of the object volume to the volume of a sphere with the same surface area, which is 1 for a sphere and gives lower values as the shape becomes less regular.

Sphericity is the ratio of the surface area of a sphere with the same volume as the object to the surface area of the object, give 1 for a sphere and lower values as the shape becomes less compact.

Shape Descriptor	Formula
Surface Area:Volume	$\mathrm{SA{:}V} = \frac{\mathrm{SA}}{\mathrm{V}}$
Radius Variance	$r(j) = \sqrt{(c_x - p(j)_x)^2 + (c_y - p(j)_y)^2 + (c_z - p(j)_z)^2}$ Radius variance = $\frac{\sum_{j=0}^{n}(r(j)-\bar{r})^2}{n}$
Compactness	$d(k) = \sqrt{(c_x - p(k)_x)^2 + (c_y - p(k)_y)^2 + (c_z - p(k)_z)^2}$ $\text{Compactness} = \frac{\left(\frac{\sum_{k=0}^{n}(d(k)-\bar{d})^2}{n}\right)}{V}$
Distribution Isotropy	$d(k)_x = \sqrt{(c_y - p(k)_y)^2 + (c_z - p(k)_z)^2}$ $d(k)_y = \sqrt{(c_x - p(k)_x)^2 + (c_z - p(k)_z)^2}$ $d(k)_z = \sqrt{(c_y - p(k)_y)^2 + (c_x - p(k)_x)^2}$ $\text{Isotropy} = \frac{\left(\frac{\sum_{k=0}^{n}(d(k)_x-\overline{d_x})^2}{n}\right)}{\left(\frac{\sum_{k=0}^{n}(d(k)_y-\overline{d_y})^2}{n}\right)} + \frac{\left(\frac{\sum_{k=0}^{n}(d(k)_x-\overline{d_x})^2}{n}\right)}{\left(\frac{\sum_{k=0}^{n}(d(k)_z-\overline{d_z})^2}{n}\right)} + \frac{\left(\frac{\sum_{k=0}^{n}(d(k)_y-\overline{d_y})^2}{n}\right)}{\left(\frac{\sum_{k=0}^{n}(d(k)_z-\overline{d_z})^2}{n}\right)}$
Isoperimetric quotient	$\mathrm{IPQ} = \frac{V}{\frac{4}{3}\pi\left(\frac{A}{4\pi}\right)^3}$
Sphericity	$\text{Sphericity} = \frac{\pi^{\frac{1}{3}}6V^{\frac{2}{3}}}{A}$

V = object volume
A = object surface area
n = number of voxels making up the object
$p(k)$ = object voxel k
$d(k)$ = distance of object voxel k from the object centroid
$d(k)_s$ = distance of object voxel k from axis s
$r(j)$ = distance of object surface voxel j from the object centroid

References

1. Schmid B, Schindelin J, Cardona A, Longair M, Heisenberg M: A high-level 3D visualization API for Java and ImageJ. *BMC Bioinformatics* 2010, 11:274.
2. Otsu, N. A Threshold Selection Method from Gray-Level Histograms. *IEEE Trans. Syst. Man Cybern.* **9,** 62–66 (1979).
3. Mullikin, J. C. & Verbeek, P. W. Surface area estimation of digitized planes. *Bioimaging* **1,** 6–16 (1993).

License

Copyright 2014, Richard Butler, Gurdon Institute Imaging Facility.
This plugin is distributed under the terms of the GNU General Public License (https://www.gnu.org/licenses/gpl.txt) and comes with absolutely no warranty.
