# Supplementary material for: MitoLoc: A method for the simultaneous quantification of mitochondrial network morphology and membrane potential in single cells
Source: Mitochondrion. 2015 Sep;24:77–86. doi: 10.1016/j.mito.2015.07.001 (PMC4570932; doi:10.1016/j.mito.2015.07.001)

Supplementary material

Supplementary Fig. 1: Intensity correlation scatter plot for wild type and respiration-deficient ρ^0 cells. Broken lines represent a least squares linear regression fit with $r^2 = 0.87$ and $r^2 = 0.31$, respectively.

Supplementary Fig. 2: A sample number as low as 6 is sufficient to obtain statistically significant data.

Supplementary Fig. 3: Mitochondrial membrane potential is not affected by treatment with H_2O_2, as measured by $DiOC_6$ accumulation. $1x10^6$ wild-type cells were harvested and incubated with medium, medium containing 1.0 mM H_2O_2 or medium with 15 μM CCCP for 45 min. Then, cells were stained for 15 min with 175 nM $DiOC_6$ in 10 mM HEPES 5 % glucose, washed and examined using the FITC filter set.

Supplementary Fig. 1

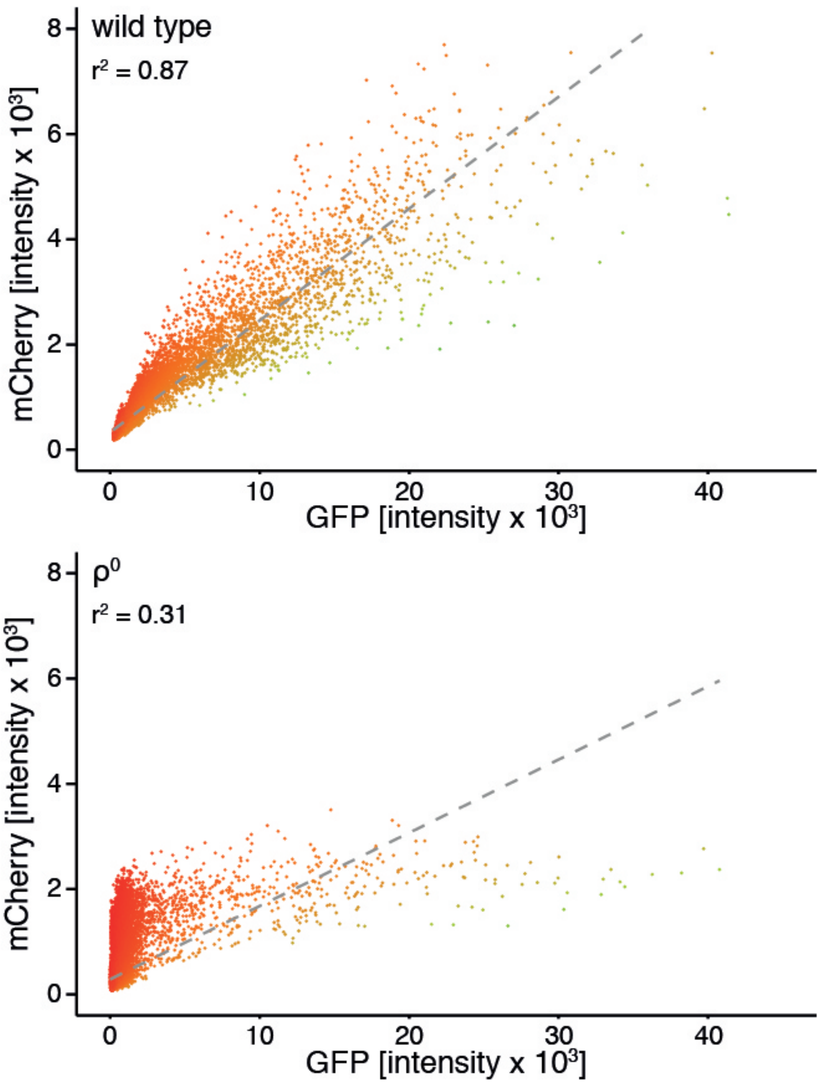

Supplementary Fig. 2

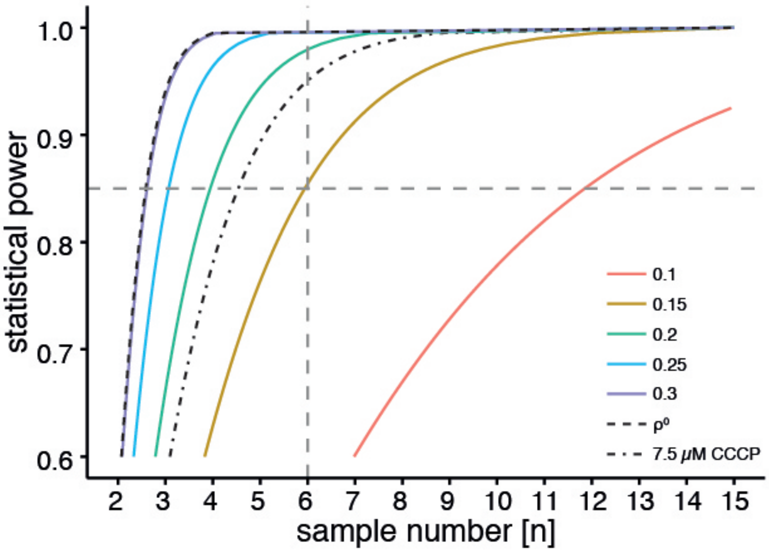

Supplementary Fig. 3

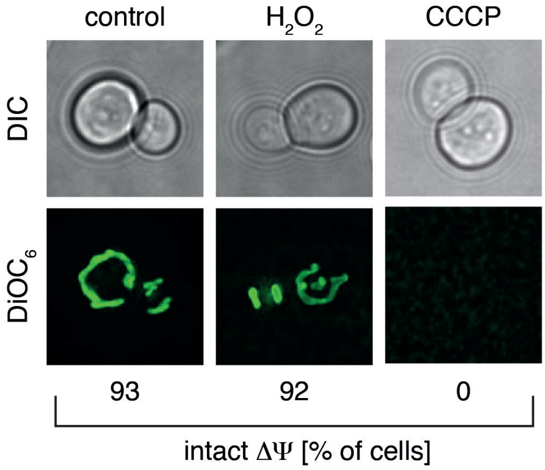

Supplement: Supplementary file 2 — Supplementary figures. [file mmc2.pdf]
